# Management of Epstein–Barr Virus Infection and Post-Transplant Lymphoproliferative Disorder in Pediatric Liver Transplantation

**DOI:** 10.3390/jcm11082166

**Published:** 2022-04-13

**Authors:** Tatsuya Okamoto, Hideaki Okajima, Elena Yukie Uebayashi, Eri Ogawa, Yosuke Yamada, Katsutsugu Umeda, Hidefumi Hiramatsu, Etsurou Hatano

**Affiliations:** 1 Department of Pediatric Surgery, Kyoto University Hospital, Kyoto 606-8507, Japan; ho1962@kuhp.kyoto-u.ac.jp (H.O.); elenayy@kuhp.kyoto-u.ac.jp (E.Y.U.); erio24@kuhp.kyoto-u.ac.jp (E.O.); etsu@kuhp.kyoto-u.ac.jp (E.H.); 2 Department of Surgery, Kyoto University Graduate School of Medicine, Kyoto 606-8507, Japan; 3 Department of Pediatric Surgery, Kanazawa Medical University Hospital, Kanazawa 920-0265, Japan; 4 Department of Diagnostic Pathology, Kyoto University Hospital, Kyoto 606-8507, Japan; yyamada@kuhp.kyoto-u.ac.jp; 5 Department of Pediatrics, Kyoto University Hospital, Kyoto 606-8507, Japan; umeume@kuhp.kyoto-u.ac.jp (K.U.); hiramatu@kuhp.kyoto-u.ac.jp (H.H.)

**Keywords:** Epstein–Barr virus (EBV), post-transplant lymphoproliferative diseases (PTLD), liver transplantation, pediatric

## Abstract

With the advancement of immunosuppressive strategies, the outcome of liver transplantation during childhood has dramatically improved. On the other hand, Epstein–Barr virus (EBV) infection and associated post-transplant lymphoproliferative diseases (PTLD), such as malignant lymphoma, are serious complications that contribute to morbidity and mortality, and are still an important issue today. Recently, an early diagnosis by quantitative PCR and PET-CT testing, and treatment with rituximab (an anti-CD20 antibody) has been established, and long-term remission has been achieved in many cases. However, the optimal immunosuppression protocol after remission of PTLD needs to be determined, and it is hoped that a treatment for refractory PTLD (e.g., PTL-NOS) will be proposed.

## 1. Introduction

With the advancement of immunosuppressive agents and strategies, the outcomes of liver transplantation have dramatically improved. In particular, the 5-year survival rate after liver transplantation for pediatric liver disease now exceeds 90% [1]. However, chronic complications, such as renal failure, infection, and malignant neoplasms associated with immunosuppression remain a problem. In particular, Epstein–Barr virus (EBV) infection causes serious complications that contribute to morbidity and mortality, such as post-transplant lymphoproliferative diseases (PTLD), especially malignant lymphoma. In this paper, we describe the clinical features of PTLD, and outline the management of EBV infection and PTLD after pediatric liver transplantation according to the experience of our institution. This case review was approved by the Institutional Review Board of Kyoto University Hospital, Japan (R-1473-3).

## 2. The Pathogenesis of Epstein–Barr Virus

EBV is one of the eight types of human herpes viruses, and is a double-stranded linear DNA virus that was discovered by Epstein et al. in 1964 in cultured Burkitt’s lymphoma cells. EBV infects naïve B cells in the pharynx via saliva [2,3]. Most individuals are asymptomatically infected in childhood, and more than 95% of adults show a previous infection pattern [4]. If the initial infection occurs during adolescence, when cellular immunity is not yet established, infectious mononucleosis develops due to transient proliferation of infected B lymphocytes. After the initial infection is over, EBV latently infects some memory B cells and the infected B cells become immortalized, but their proliferation is controlled by EBV-specific cytotoxic T lymphocytes (CTLs) [5]. However, in the state of cellular immunodeficiency associated with immunosuppression during solid organ transplantation (SOT) or allogenic hematopoietic stem cell transplantation (allo-HSCT), EBV-infected B cells evade cellular immunity and begin to proliferate again. B cell-derived PTLD can develop through this mechanism.

EBV latently infects host cells and the full-length viral genome is maintained as a plasmid, and the expression pattern of viral genes and proteins changes depending on the cell type and the condition of the latently infected cell (Table 1) [6]. Latent infection can be divided into four types: types 0, I, II, and III. Latent infection type 0 is seen when the memory B cells of healthy individuals are infected and express only EBV-encoded RNAs (EBERs). In addition to EBERs, type I expresses a group of genes called BamHI A rightward transcripts (BARTs) and the antigen of EBV nuclear antigen (EBNA)-1. Type II expresses latent membrane protein (LMP) 1, LMP2A, and LMP2B. Type III expresses all latent associated proteins (e.g., EBNA-2, -3A, -3B, -3C, and –LP), and is more proliferative [6]. In addition to B cells, EBV infects T cells, NK cells, and epithelial cells, and is known to be associated with various diseases. However, the mechanism of infection is still unknown.

## 3. Post-Transplant Lymphoproliferative Diseases (PTLD)

PTLD is a general term for lymphoproliferative diseases and lymphomas that develop after SOT or allo-HSCT. A distinction should be made between PTLD after SOT and PTLD after allo-HSCT in that PTLD after SOT is mainly derived from recipient cells whereas PTLD after allo-HSCT is mainly derived from donor cells [6]. The incidence of PTLD in SOT is 13–33% in multiple visceral transplantation, 7–11% in small bowel transplantation, 9.4% in heart and lung transplantation, 1.8–7.9% in lung transplantation, 3.4% in heart transplantation, 2.2% in liver transplantation, and 1% in kidney transplantation [7].

Risk factors for the development of PTLD after SOT include the use of calcineurin inhibitors (CNIs), pediatric transplantation, transplantation from an EBV seropositive donor to a seronegative recipient, previous cytomegalovirus (CMV) infection, and the use of anti-human thymocyte immunoglobulin [8,9,10]. The most common time of onset is within one year after transplantation [10]. However, it should be noted that the onset of the disease may occur after 10 years due to the lifelong use of immunosuppressive drugs in SOT. Common clinical symptoms include upper respiratory tract symptoms, enlarged tonsils, lymphadenopathy, and abdominal symptoms such as abdominal pain, diarrhea, and bloody stool [10]. In addition, there are cases of acute abdomen, including abdominal masses, intestinal obstruction, and perforation.

PTLD includes a variety of pathological conditions ranging from benign (polyclonal) to malignant (monoclonal). The disease is classified by WHO according to the pathologic morphology and clonality, as shown in Table 2 [11]. Most tumor tissues are derived from EBV-positive B cells, clearly indicating that EBV plays a major role in the pathogenesis of PTLD, whereas T/NK cell-derived PTLD is often negative for EBV. EBV-associated T/NK-PTLD occurs in approximately 10% of all PTLD, and 85% of these cases occur within the first year after transplantation [12]. On the other hand, EBV-negative T/NK-PTLD often develops long after transplantation and has been reported to occur as late as 5 years after transplantation [13].

## 4. Diagnosis of PTLD

In principle, the diagnosis of PTLD depends on pathological examination; however, clinical symptoms, blood tests, and imaging studies are also considered in a comprehensive manner. As mentioned above, since the EVB infection status is associated with the risk of developing PTLD, recipients and donors should undergo screening for EB VCA-IgG and EBNA antibodies, such as serological tests for EBV, prior to transplantation. In addition, monitoring of EBV-DNAemia by quantitative PCR is useful for early detection of EBV-positive PTLD [14]. We regularly measure EBV-DNA in patients after pediatric liver transplantation, once a week in the perioperative period and once every 1–3 months after hospital discharge. If the blood EBV-DNA load reaches ≥1.0×10^3^ copies/μg DNA during the course of the disease, we conduct a thorough examination, including imaging tests, as described below. In the case of unexplained cytopenia, increased LDH, hypoalbuminemia, high transaminases, hyperuricemia, or other blood test abnormalities PTLD should be suspected. Currently, there are no established biomarkers for use in EBV positive PTLD [15]; however, the measurement of soluble interleukin-2 receptor (sIL2R) and serum ferritin levels as tumor markers may assist in the diagnosis.

When PTLD is suspected, computerized tomography (CT) or magnetic resonance imaging (MRI) of the head and whole body is generally performed to determine the site of the lesion, including lymphadenopathy and mass formation. As with other malignancies, screening by positron emission tomography-CT (PET-CT) is useful for the detection of PTLD [9,10,14]. In cases with gastrointestinal symptoms such as diarrhea or bloody stool, gastrointestinal endoscopy should be performed to determine the site of the lesion and biopsy should be performed. In cases with central nervous system symptoms, head MRI and spinal fluid test are indicated, and in cases with suspected bone marrow infiltration, bone marrow examination is indicated.

As mentioned previously, a pathological examination is essential for the definitive diagnosis of PTLD, and the diagnosis is made according to the 2016 revised WHO classification of leukemia and lymphoid tumors [11]. An immunological search (identification of T and B cells) and search for EBV (EBER in situ hybridization; EBER-ISH, LMP, and EBNA) are also performed. In addition, a portion of the biopsy specimen will be divided into cell suspensions for immunological phenotyping by flow cytometry, chromosome testing (chromosome G-banding) and genetic testing (BCL-6 mutation, RAS mutation, TP53 mutation, MIC mutation, and immune-related gene (IGH, IGK, and IGL TCR) reconstruction).

**Figure 1:**
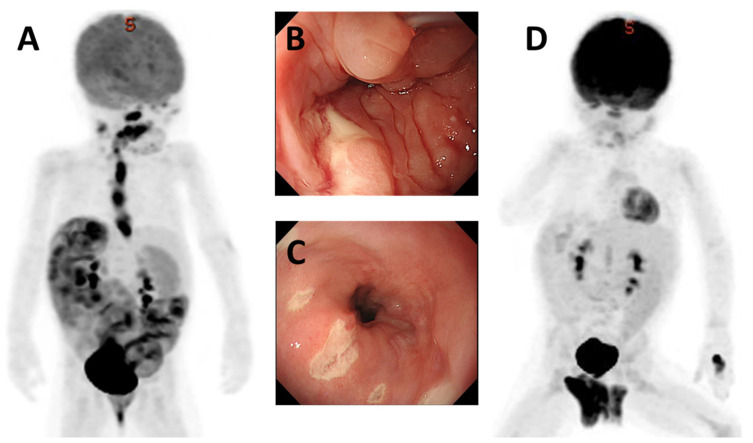
EBV-positive mucocutaneous ulcer (EBVMCU). Case 1. A girl of 1 year and 5 months of age. At 8 months after LDLT, she developed gastrointestinal symptoms, including abdominal pain and watery stool, with fever. (**A**) PET-CT showed numerous hot spots, especially in the esophagus. (**B**,**C**) Upper endoscopy revealed numerous ulcerative lesions in the esophagus, and biopsy revealed EBVMCU. (**D**) Her symptoms resolved within 2 weeks after dose reduction of her immunosuppressive drugs, and PET-CT showed the disappearance of the hot spots of the gastrointestinal tract.

As an example of the diagnosis, we herein describe a case of EBV-associated esophagitis after liver transplantation in our institution. The patient was 1 year and 5 months of age at the time of her diagnosis. She underwent living donor liver transplantation (LDLT) with her father as the donor at 7 months of age due to biliary atresia. There was an episode of severe acute rejection in the perioperative period, which required the administration of thymoglobulin and maintenance with four immunosuppressive drugs (tacrolimus (FK), mycophenolate mofetil (MMF), prednisolone (PSL), and everolimus (EVL)). At 8 months postoperatively, she developed gastrointestinal symptoms, including abdominal pain and watery stool, with fever. The EBV-DNA load increased to 2300 copies/μg DNA. Simultaneously, her sIL2R value increased to 2810U/mL, and PET-CT showed numerous hot spots, especially in the esophagus (Figure 1A). Upper endoscopy revealed numerous ulcerative lesions in the esophagus (Figure 1B,C), and biopsy revealed an EBV-positive mucocutaneous ulcer (EBVMCU).

In this case, all immunosuppressive drugs other than PSL were discontinued, and the patient’s symptoms rapidly resolved within 2 weeks; however, at the same time, her transaminase level increased, and liver biopsy revealed late-onset acute T-cell mediated rejection. At 2 weeks after the diagnosis, PET-CT showed that the hot spot had disappeared and her EBV-DNA load had decreased to 100 copies/μg DNA (Figure 1D); no recurrence has been observed during follow-up. As shown in this case, when EBV infection is suspected based on clinical symptoms, the prompt performance of quantification of EBV-DNA in the blood, measurement of tumor markers, and PET-CT can prevent progression to PTLD, by indicating the need to reduce the dose of immunosuppressive drugs.

## 5. Treatment of PTLD

Prophylactic therapy has been attempted for cases at high risk for PTLD; however, according to the results of a meta-analysis of antiviral prophylaxis in these high-risk patients demonstrated no benefit in relation to the development of PTLD [16]. In addition, the usefulness of preemptive treatment with an anti-CD20 antibody preparation (rituximab) in allo-HSCT patients with an increased blood EBV-DNA load has been recognized [17]; however, the usefulness in SOT is unknown.

The first line of treatment for PTLD is reduction or discontinuation of immunosuppressive drugs, especially CNIs [6,7,9,10,14,18]. On the other hand, because of the increased risk of acute rejection, during dose reduction, blood tests must be frequently performed in order to detect deviation of enzyme levels. In recent years, everolimus (EVL), an mTOR inhibitor, has been reported to inhibit the growth of EBV-infected cells in vitro [19], and EVL may be used in combination with the reduction of CNIs.

Rituximab used in the treatment of malignant lymphoma is also useful for CD20-positive PTLD; thus, combination therapy with chemotherapy is the treatment of choice for monomorphic PTLD. For non-Hodgkin’s lymphoma, rituximab plus CHOP (R-CHOP) has been found to have better results than CHOP, and R-CHOP is also the treatment of choice for PTLD [20]. For classical Hodgkin lymphoma, involved-field irradiation may be considered in addition to chemotherapy [21].

## 6. Experience of PTLD in Our Institution

From January 1990 to April 2019, 923 patients under 18 years of age underwent liver transplantation in our department. Forty-five of these patients were pathologically diagnosed with PTLD. Our cases are summarized in Table 3. There were 30 cases of non-destructive PTLD (NP), 2 cases of polymorphic PTLD (PP), and 13 cases of monomorphic PTLD (MP). The pathological diagnosis of the 13 MP cases was DLBCL (n = 8), Burkitt (n = 3), PTL-NOS (n = 1), and Pre-B ALL (n = 1). The median time from transplantation to the diagnosis of PTLD was 14 months (interquartile range (IQR) 4-32), and when limited to MP, the median time tended to be slightly longer, at 30 months (IQR 7-53).

In our institution, since 1999, we have been routinely measuring EBV-DNA levels in blood by quantitative PCR. We therefore divided these 45 cases into two groups: early period cases (from 1990 to 1998) and late period cases (from 1999 to 2019). In the early period, PTLD was diagnosed in 28 of 362 transplanted patients, including NP (n = 20), PP (n = 2), and MP (n = 6), accounting for 7.7% of all transplanted patients, whereas in the late period, PTLD was diagnosed in 17 of 551 transplanted patients, including NP (n = 10) and MP (n = 7). The incidence decreased to 3.0%.

In addition, the prognosis of patients in the early period was as follows: tumor-related death occurred in three of the six cases of MP, and two patients achieved remission of PTLD but subsequently died of graft liver dysfunction. Death occurred in 1 of 2 PP cases and 7 of the 20 NP cases. Five of these deaths were due to eventual graft dysfunction associated with poor control of rejection, one was due to cerebral hemorrhage, and the other was due to a traffic accident. On the other hand, in the late period cases, three of the seven patients with MP died (all were tumor-related deaths; one of these patients developed PTLD (DLBCL) after small bowel liver transplantation). In addition, 1 of the 10 patients with NP died of GVHD after liver transplantation; however, the other cases remain alive. All patients were treated by reducing or stopping immunosuppressive drugs, and one patient with early-stage disease was treated with rituximab twice because of residual lymph node enlargement after the cessation of immunosuppressive drugs.

According to the review of cases in our institution, the incidence of PTLD decreased after the introduction of EBV-DNA quantitative testing; however, another problem was realized in that PTLD extended not only to lymphoma (i.e., monomorphic PTLD), but also to early-stage lesions of non-destructive PTLD that were difficult to control with subsequent immunosuppression and infection. Many patients were eventually lost due to graft cirrhosis. Since the introduction of quantitative EBV-DNA testing, monitoring of EBV has become possible, and it has become relatively easy to maintain a balance between infection and rejection, which has improved the prognosis of early-stage disease. However, the question of when to resume immunosuppression and at what dose after PTLD remission is still an issue that needs to be addressed to improve long-term survival. To illustrate this, we present a case that was managed at our department.

**Figure 2:**
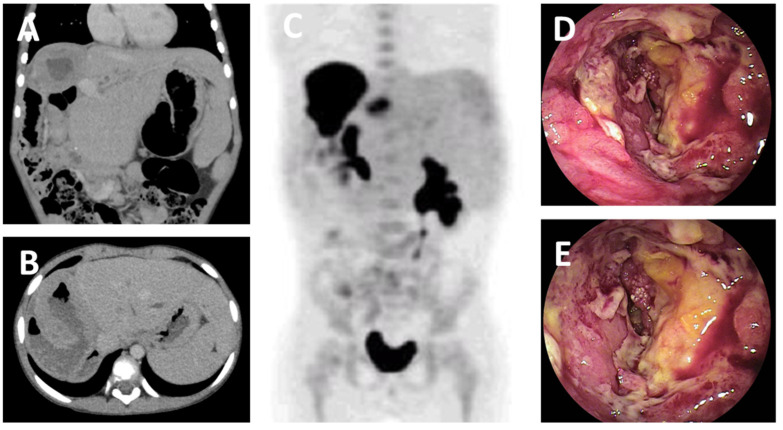
Monomorphic PTLD; DLBCL. Case 2. A boy of 6 years and 6 months of age. At 4 years and 5 months after LDLT, he was readmitted to our hospital with abdominal pain, fever, and vomiting. (**A**,**B**) The initial CT examination revealed thickened enlargement of the Roux-en Y jejunum. (**C**) PET-CT confirmed the accumulation of radionuclides in the same area. (**D**,**E**) Small bowel endoscopy revealed mucosal erosion and wall thickening. Biopsy specimens were obtained.

**Figure 3:**
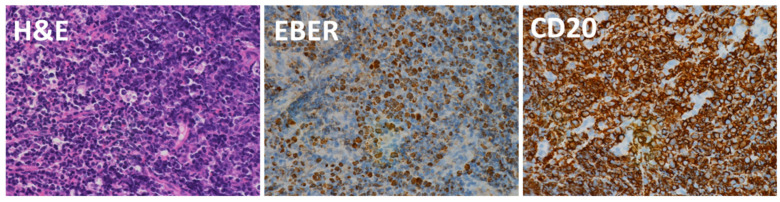
DLBCL. Monomorphic B-cell PTLD; DLBCL, showing diffuse infiltration of atypical lymphocytes with numerous apoptosis. These lymphocytes are positive for CD20 and EBER.

The patient was 6 years and 6 months of age at the time of the diagnosis of PTLD. He underwent LDLT (compatible) with his father as the donor at 2 years and 1 month of age due to biliary atresia. The patient had multiple episodes of acute rejection and was on 3 immunosuppressive drugs (FK, MMF, and PSL). His postoperative blood EBV-DNA load remained low at 10–50 copies/µg DNA. At four years and five months after surgery, the patient was readmitted to our hospital with abdominal pain, fever, and vomiting, and the initial CT examination revealed thickened enlargement of the Roux-en Y jejunum (Figure 2A,B), while PET-CT confirmed the accumulation of radionuclides in the same area (Figure 2C). Biopsy specimens were obtained by small bowel endoscopy (Figure 2D,E). Pathological findings were shown in Figure 3. Monomorphic B-cell PTLD, showing diffuse infiltration of atypical lymphocytes with numerous apoptosis. These lymphocytes are positive for CD20 and EBER, and a diagnosis of DLBCL was made.

**Figure 4:**
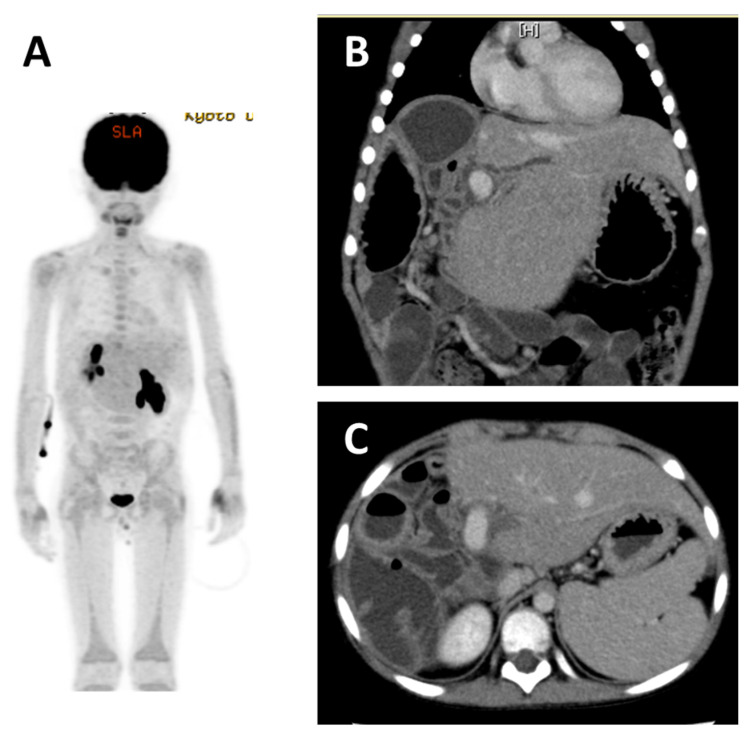
Monomorphic PTLD; DLBCL, post-chemotherapy. Case 2 (continued). Among the immunosuppressive drugs, only tacrolimus was discontinued, and he was treated with rituximab plus B-NHL03. Complete remission was achieved 8 months after the start of treatment. (**A**) PET-CT confirmed no accumulation of radionuclides. (**B**,**C**) CT examination revealed improvement of thickened enlargement of the Roux-en Y jejunum.

Among the immunosuppressive drugs, only FK was discontinued, and the patient was treated with rituximab plus Japanese B-NHL03 protocol chemotherapy. Complete remission was achieved 8 months after the start of treatment (Figure 4). Liver biopsy after the end of treatment suggested T-cell-related rejection; thus, FK was restarted at 1 year after the start of treatment. To date, there has been no recurrence of PTLD and no acute rejection; however, a tendency toward atrophy of the interlobular bile ducts has been observed, which suggests early chronic rejection. The strategy for immunosuppression after the remission of PTLD should be investigated in a multi-center study in the future.

We only experienced one case of EBV-associated T/NK cell tumor; however, the tumor progressed rapidly and was difficult to control. A summary is presented below. 

**Figure 5:**
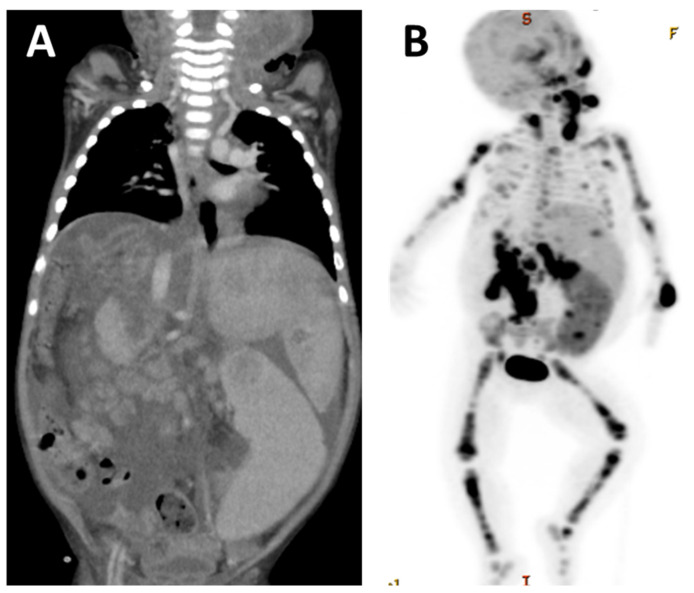
Monomorphic PTLD; PTL-NOS. Case 3. A girl of 1 year and 6 months of age. She received LDLT at 11 months of age due to biliary atresia. At 7 months after surgery, she was admitted to the hospital due to fever and enlargement of her left posterior cervical lymph nodes. (**A**) The initial CT scan revealed left cervical lymphadenopathy and multiple enlarged abdominal lymph nodes, and mass formation was observed in the spleen. (**B**) PET-CT showed the diffuse accumulation of radionuclides in the lymph nodes, bones, and spleen.

**Figure 6:**
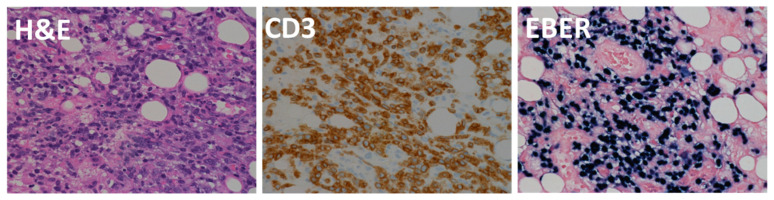
PTL-NOS, Cervical lymph node biopsy revealed monomorphic T-cell PTLD. Peripheral T-cell lymphoma, NOS, showing atypical lymphoid cells with irregular nuclei. They are positive for CD3 and EBER.

The patient was a girl of 1 year and 6 months of age. She received LDLT at 11 months of age due to biliary atresia. The patient was discharged from the hospital on the 56th postoperative day, and immunosuppression with FK + PSL was continued. At 3 months after surgery, her EBV-DNA load increased to 2300 copies/µg DNA, and the patient was carefully examined by CT, but no lymph node enlargement was noted. The patient was followed up with the dose reduction of her immunosuppressive drugs; however, at 7 months after surgery, she was admitted to our hospital for fever and enlarged left posterior cervical lymph nodes. Her EBV-DNA load increased to 5200 copies/µg DNA, her sIL2R value was 4660 U/mL, and PET-CT showed numerous lymph nodes and hot spots in the bone, spleen, and cervical lymph nodes (Figure 5A,B). Cervical lymph node biopsy revealed monomorphic PTLD; PTL-NOS (Figure 6). Chemotherapy with CHOP was started, but the rapid progression of the disease could not be controlled and the patient died 9 months after transplantation.

## 7. Conclusions

In the past, PTLD was a fatal disease with no effective treatment once it progressed; however, with the establishment of an early diagnosis of EBV-DNAemia by quantitative PCR and PET-CT and the availability of treatment with rituximab, remission can now be achieved in many patients. In order to further improve the results, it is necessary to investigate how to achieve optimal immunosuppression after the remission of PTLD, as shown in Case 2. In addition, as shown in Case 3, there is still no effective treatment for cases in which the EBV host is T cells, and it is difficult to save the lives of such patients. Considering that not all children experience EBV infection after liver transplantation, in order to improve treatment outcomes in the future, it would be desirable to identify the factors that determine the affinity of immune cells for EBV infection and to elucidate the molecular mechanisms that are clinically valid therapeutic targets.

## Figures and Tables

**Table 1:** The different viral latency stages and EBV gene and encoded protein expression patterns.

EBV Genes and Encoded Proteins		Latency			
		Type 0	Type I	Type II	Type III
RNAs	EBERs	+	+	+	+
	BARTs	±	+	+	+
Proteins	EBNA-1	−	+	+	+
	EBNA-2	−	−	−	+
	EBNA-3s	−	−	−	+
	EBNA-LP	−	−	−	+
	LMP-1	−	−	+	+
	LMP-2	−	−	+	+

**Table 2:** The 2016 revised WHO classification of PTLDs.

I. Non-destructive PTLD
Plasmacytic hyperplasia
Infectious mononucleosis
Florid follicular hyperplasia
II. Polymorphic PTLD
III. Monomorphic PTLD
a B-cell neoplasms
Diffuse large B-cell lymphoma (DLBCL)
Burkitt’s lymphoma
Plasma cell myeloma
Others
b T/NK cell neoplasms
Peripheral T-cell lymphoma not otherwise specified (PTL-NOS)
Others
IV. Classic Hodgkin lymphoma PTLD

**Table 3:** Summary of PTLD cases.

	Years		Years	
Classification	1990–1998 Total	Alive	1999–2019 Total	Alive
I. Non-destructive PTLD	30	23	10	9
II. Polymorphic PTLD	2	1		
III. Monomorphic PTLD				
a B-cell neoplasms				
Diffuse large B-cell lymphoma (DLBCL)	4	0	4	3
Burkitt’s lymphoma	1	0	2	1
Plasma cell myeloma				
Others	1	1		
b T/NK cell neoplasms				
Peripheral T-cell lymphoma not otherwise specified (PTL-NOS)			1	0
Others				
IV. Classic Hodgkin lymphoma PTLD				

## Data Availability

The data that support the findings of this study are available from the corresponding author, (T.O.), upon reasonable request.

## Abbreviations

allo-HSCT, allogeneic hematopoietic stem cell transplantation; BARTs, BamHI A rightward transcripts; CMV, cytomegalovirus; CNIs, calcineurin inhibitors; CT, computerized tomography; CTLs, cytotoxic T lymphocytes; EBV, Epstein–Barr virus; EBERs, EBV-encoded RNAs; PTLD, post-transplant lymphoproliferative disorder; EVL, everolimus; EVNA, EBV nuclear antigen; FK, tacrolimus; LDLT, living donor liver transplantation; LMP, latent membrane protein; MMF, mycophenolate mofetil; MP, monomorphic PTLD; MRI, magnetic resonance imaging; NP, non-destructive PTLD; PET, positron emission tomography; PP, polymorphic PTLD; PSL, prednisolone; sIL2R, soluble interleukin-2 receptor; SOT, solid organ transplantation.
